# Unravelling the diversity in water usage among wild banana species in response to vapour pressure deficit

**DOI:** 10.3389/fpls.2023.1068191

**Published:** 2023-08-21

**Authors:** David Eyland, Clara Gambart, Rony Swennen, Sebastien Carpentier

**Affiliations:** ^1^ Laboratory of Tropical Crop Improvement, Division of Crop Biotechnics, KU Leuven, Heverlee, Belgium; ^2^ International Institute of Tropical Agriculture, Banana Breeding, Kampala, Uganda; ^3^ Bioversity International, Biodiversity for Food and Agriculture, Leuven, Belgium

**Keywords:** drought tolerance, stomatal conductance, transpiration, vapour pressure deficit, water use efficiency, wild banana species, breeding

## Abstract

The rise in global temperature is not only affecting plant functioning directly, but is also increasing air vapour pressure deficit (VPD). The yield of banana is heavily affected by water deficit but so far breeding programs have never addressed the issue of water deficit caused by high VPD. A reduction in transpiration at high VPD has been suggested as a key drought tolerance breeding trait to avoid excessive water loss, hydraulic failure and to increase water use efficiency. In this study, stomatal and transpiration responses under increasing VPD at the leaf and whole-plant level of 8 wild banana (sub)species were evaluated, displaying significant differences in stomatal reactivity. Three different phenotypic groups were identified under increasing VPD. While (sub)species of group III maintained high transpiration rates under increasing VPD, *M. acuminata* ssp. e*rrans* (group I), *M. acuminata* ssp. *zebrina* (group II) and *M. balbisiana* (group II) showed the highest transpiration rate limitations to increasing VPD. In contrast to group I, group II only showed strong reductions at high VPD levels, limiting the cost of reduced photosynthesis and strongly increasing their water use efficiency. *M. acuminata* ssp. *zebrina* and *M. balbisiana* thus show the most favourable responses. This study provides a basis for the identification of potential parent material in gene banks for breeding future-proof bananas that cope better with lack of water.

## 1 Introduction

Climate change projections predict that global temperatures will continue to increase this century (IPCC et al., 2021). This temperature rise is not only affecting plant functioning directly, but is also increasing air vapour pressure deficit (VPD) (Hatfield and Prueger, 2015; Ficklin and Novick, 2017; Grossiord et al., 2020). VPD represents the atmospheric water vapour demand and is defined as the difference between the saturation and actual vapour pressure in the atmosphere (Monteith and Unsworth, 2013). The saturation vapour pressure, the water vapour that air can hold, increases exponentially with temperature and has been increasing as global temperatures rise (Lawrence, 2005). The actual vapour pressure (i.e. absolute humidity in the air) on the other hand has not been rising at the same rate as the saturation vapour pressure, therefore increasing the worldwide VPD (Ficklin and Novick, 2017; Grossiord et al., 2020). The impact of this rising VPD is often underestimated compared to other climate change consequences, but periods of high VPD have recently been linked with large-scale tree mortality (Breshears et al., 2013; Williams et al., 2013) and strong yield reductions (Challinor and Wheeler, 2008; Lobell et al., 2013).

Plants respond to the vapour pressure deficit encountered at the leaf level, the leaf-to-air vapour pressure deficit (VPD_leaf_). The leaf temperature can after all deviate from that of the ambient air by transpirational cooling or heating through radiant energy. For a given stomatal opening, transpiration would increase linearly with VPD_leaf_, without any gain in carbon uptake. Stomatal conductance (*g_s_*) however decreases with increasing VPD_leaf_, avoiding excessive water loss, but restricting carbon uptake (Dai et al., 1992; Monteith, 1995; Oren et al., 1999). In angiosperms the reduction of *g_s_* in response to an increase in VPD_leaf_ is believed to be abscisic acid (ABA) mediated (Xie et al., 2006; Bauer et al., 2013; McAdam and Brodribb, 2015). Upon an increase in VPD_leaf_, g*_s_* is reduced by a rapid ABA biosynthesis (i.e. within 20 min) presumably located in the leaf phloem parenchyma cells and stomatal guard cells (Kuromori et al., 2014; McAdam et al., 2016). The trigger for ABA interference under high VPD_leaf_ is believed to be a drop in water status (McAdam and Brodribb, 2016; Sack et al., 2018), which has been linked to a limited maximal hydraulic conductance at the leaf, stem and/or root level in comparison to the transpiration (Brodribb and Jordan, 2008; Zhang et al., 2013; Choudhary et al., 2014; Ocheltree et al., 2014; Schoppach et al., 2016). Essential gatekeepers for this hydraulic conductance are aquaporins. They are present all along the water transport pathway from root to stomata. Aquaporins were less abundant in soybean and pearl millet genotypes that showed a reduced transpiration rate at high VPD_leaf_ (Sadok and Sinclair, 2010; Devi et al., 2015; Reddy et al., 2017).

Despite the reductions in *g_s_*, the transpiration rate usually increases with increasing VPD_leaf_. Only at high VPD_leaf_ significant decreases in transpiration rates have been observed (Franks et al., 1997; Fletcher et al., 2007; Gholipoor et al., 2010; Ryan et al., 2016). These transpiration responses are commonly described by a segmented pattern where the slope of transpiration rate versus VPD_leaf_ is significantly reduced after a specified breakpoint. Significant differences in segmented transpiration responses to VPD_leaf_ have been observed across- and within-species (Fletcher et al., 2007; Gholipoor et al., 2010; Ryan et al., 2016). While some species or genotypes already reduce transpiration rate significantly at low VPD_leaf_, others show only a reduction at higher VPD_leaf_ or even maintain the increasing transpiration rate. Restricting transpiration rate at high VPD has been suggested as a key drought tolerance breeding trait as excessive water loss is avoided and might be saved for later in the growing season (Vadez, 2014; Sinclair et al., 2017). Limiting transpiration above a VPD threshold can increase the daily transpiration efficiency but the reduced water use may compromise the yield potential. Reduced transpiration limits carbon uptake, thereby hampering photosynthesis and yield (Richards, 2000; Lee et al., 2020; Eyland et al., 2021). Moreover, care must be taken that the so-called saved water is not merely lost by evaporation or transpiration by neighbouring plants.

The transpiration rate response to VPD was shown to be highly heritable in wheat (Schoppach et al., 2016). Models predict that in drought-prone environments limiting transpiration at high VPD would improve maize and soybean yields by maintaining more soil water available later in the season during flowering or grain filling (Sinclair et al., 2010; Messina et al., 2015). In these drought-prone regions, the negative effect of *g_s_* reduction on *A* during vegetative growth could be compensated later in the growing season (Sinclair et al., 2010; Messina et al., 2015). Improved maize hybrids which, amongst other traits, showed reduced transpiration at high VPD_leaf_ indeed increased yields under water-limited conditions (Gaffney et al., 2015), while for durum wheat cultivars this was only the case under severe drought conditions (Medina et al., 2019).

The current set of edible bananas is complex and has resulted from different parental routes and several back crosses (De Langhe et al., 2010; Perrier et al., 2011; Martin et al., 2020a; Cenci et al., 2021). The hybrid banana genomes are unbalanced with respect to the parental ones, and inter- and intra-genome translocation chromosomes are relatively common (Christelová et al., 2017; Němečková et al., 2018). Most, if not all, cultivars have genomes consisting of different proportions of A- and B-genome chromosomes and/or recombinant chromosomes originating from different parents. Similar to other tropical species, bananas are very sensitive to VPD, with reductions in transpiration when VPD exceeds 2 – 2.3 kPa (Aubert and Catsky, 1970; Carr, 2009; Eyland et al., 2022). Thomas et al. (1998) observed a diverse response in three banana cultivars with different genomic constitutions. Despite these efforts, the transpiration responses to VPD remain largely uncharacterized across diverse banana species.

Evaluation of crop wild relatives for inclusion in breeding schemes is receiving increasing attention nowadays, given their naturally acquired tolerances and resistances to biotic and abiotic stresses (Hajjar and Hodgkin, 2007; Dempewolf et al., 2017). Hence, the main objective of this work was to evaluate gene bank accessions belonging to wild banana (sub)species that can be crossed to elite edible parents. Apart from 3 unknown ancestors of the edible bananas, the *M. acuminata* (A genome) subspecies *banksii*, *zebrina*, *malaccensis* and *burmannica* form together with *M. balbisiana* (B genome) the most important parental donors of the current edible (AAA, AAB and ABB) varieties (Perrier et al., 2011; Christelová et al., 2017; Sardos et al., 2022; Martin et al., 2023). Hence, evaluating their stomatal and transpiration responses under increasing VPD at leaf and whole-plant level is of major interest to breeders. Transpiration rate limitations at high VPD have been indicated as a key breeding trait for high water use efficiency (Sinclair et al., 2010; Vadez, 2014; Messina et al., 2015; Ryan et al., 2016). Given these indications, we hypothesize that adequate stomatal reactions towards VPD is an important sub-trait to breed for drought resilient varieties and that there is intra- and interspecies variability among the banana crop wild relatives. This work could therefore provide the basis for systematically screening gene banks containing crop wild relatives of banana for their transpiration at high VPD, with the aim to identify potential parent material for drought tolerance breeding.

## 2 Materials and methods

### 2.1 Plant material & growing conditions

A diversity panel of 9 wild banana gene bank accessions belonging to 8 (sub)species (**Table 1**) were phenotyped for their transpiration response to VPD. Plants were grown in 2.5 L pots filled with peat-based compost and maintained under well-watered conditions. Plants were grown in the greenhouse for 6 - 8 weeks before moving to the growth chamber (Bronson PGC-1400, the Netherlands). The growth chamber contained an air mixing fan and LED panels providing a light intensity of 250 µmol m^-2^ s^-1^ for a 12 h photoperiod and a light spectrum with blue:red:far-red ratio of 1: 1.5: 0.15. Plants were acclimated to the growth chamber for one day under a day/night temperature and relative humidity of 27/24.5°C and 78%, respectively. The next day the VPD step-changes were initiated by altering relative humidity, while temperature was maintained at 36°C during this day. VPD was increased by decreasing relative humidity as temperature fluctuations would not only affect VPD but also aquaporin conductance and water viscosity in xylem and mesophyll cells (Matzner and Comstock, 2001; Yang et al., 2012). At light onset relative humidity was maintained for 90 min at 87%, after which it was subsequently decreased to 78, 68, 62 and 56%, each for 60 min. Average VPDs at each step were 0.77, 1.36, 1.93, 2.34 and 2.64 kPa. Plants were maintained under well-watered conditions by daily watering before light onset. Measurements were taken before 14:00 to avoid afternoon stomatal closure (van Wesemael et al., 2019; Eyland et al., 2021).

**Table 1:** Wild banana gene bank accessions screened for their transpiration response to increasing vapour pressure deficit (VPD) at both leaf and whole-plant level.

Name	Subspecies	ITC or collection code^1^	Origin ^2^	Collection site^3^	Collection coordinates^3^
Balbisiana	*Musa balbisiana*	/	Southeast China, northern Indo-Burma, Southwest India, Sri Lanka, Philippines, New Guinea	Japan (Amami)	/
Banksii_11	*Musa acuminata* ssp. *banksii*	SJP416	New Guinea	Papua New Guinea (Madang)	5° 37’ 8” S 145° 28’ 7” E
Banksii_17	*Musa acuminata* ssp. *banksii*	SJP814	New Guinea	Papua New Guinea (Morobe)	6° 44’ 42” S 146° 43’ 51” E
Burmannica	*Musa acuminata* ssp*. burmannica*	ITC0283	southern Indo-Burma	/	/
Burmannicoide*s*	*Musa acuminata* ssp. *burmannicoides*	ITC0249	southern Indo-Burma	/	/
Errans	*Musa acuminata* ssp. *errans*	ITC1028	Philippines	/	/
Malaccensis_33	*Musa acuminata* ssp. *malaccensis*	928533	Sumatra and Malayan Peninsula	Malaysia (Pahang)	3°53’51” N 102°12’23”E
Microcarpa	*Musa acuminata* ssp. *microcarpa*	ITC0253	Borneo	/	/
Zebrina	*Musa acuminata* ssp*. zebrina*	ITC1177	Sumatra and Malayan Peninsula	/	/

^1^Accessions without ITC code are not yet publicly available at the International Transit Centre (ITC) gene bank. The collection code represents the given code to the mother plant during collection. ^2^Accession origin as described by Janssens et al. (2016). ^3^Only locations of collected samples are shown.

### 2.2 Leaf gas exchange measurements

**Figure 1:**
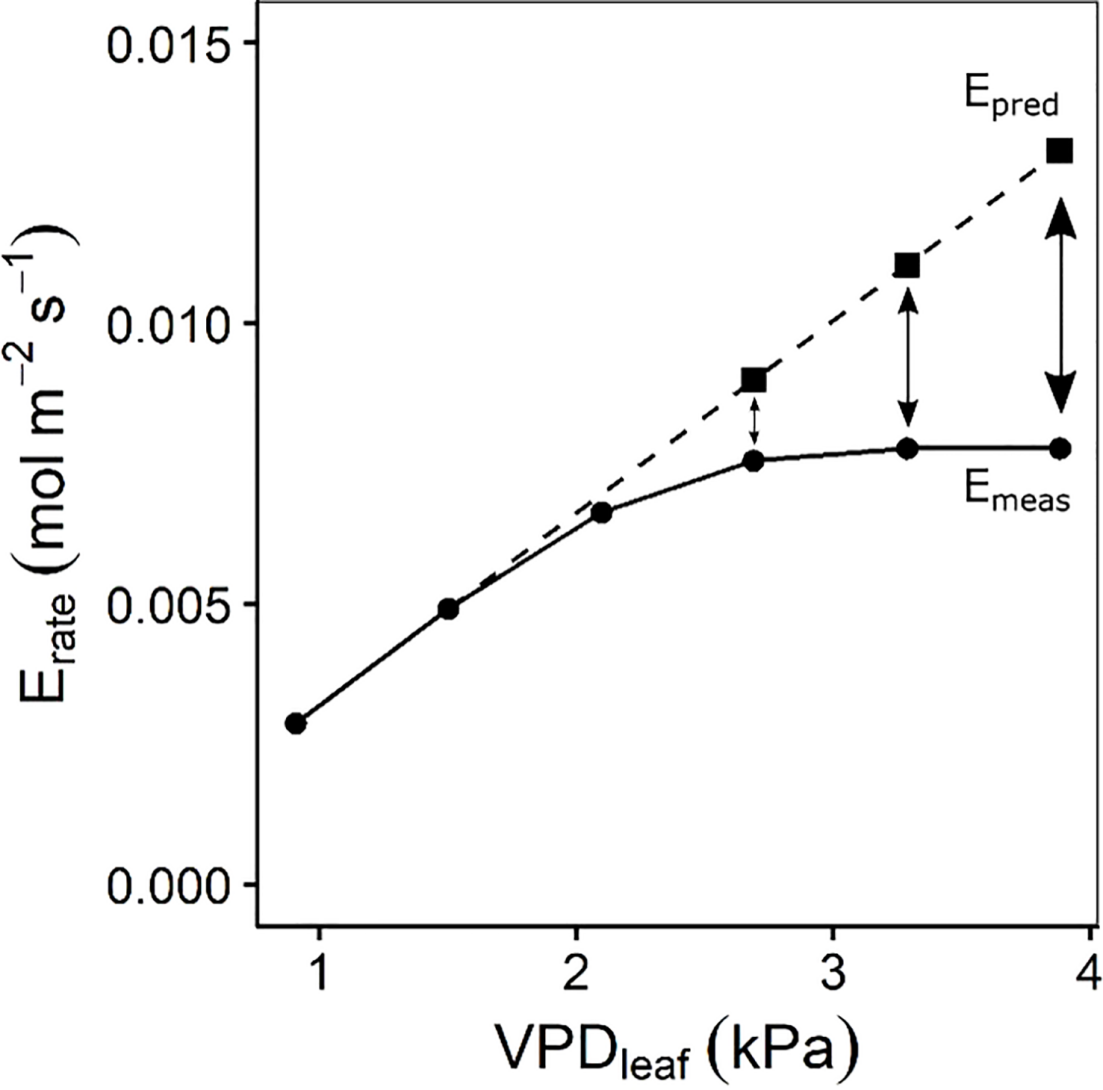
Quantification of the transpiration reduction (ϕ_E_) according to Franks et al (1999). and Ryan et al. (2016). A linear regression was fitted through the transpiration rate at the first two air-to-leaf vapour pressure deficit (VPD_leaf_) levels. This linear regression was extrapolated (dashed line) to estimate the transpiration rate (E_pred_) at VPD_leaf_ of 2.69, 3.28 and 3.87 kPa. E_pred_ was then compared to the measured transpiration rate (E_meas_) to calculate ϕ_E_ (Eq. 1).


Gas exchange responses to step increases in VPD_leaf_ were measured every 60 s on the middle of the second youngest fully developed leaf using a LI-6800 infrared gas analyser (LI-COR, USA). Light intensity and CO_2_ concentration were maintained at 250 µmol m^-2^ s^-1^ and 400 µmol mol^-1^, respectively. Leaf temperature was maintained at 36°C. Relative humidity went from 85 to 75, 65, 55, 45 and 35%, reaching VPD_leaf_ of 0.91, 1.50, 2.09, 2.69, 3.28 and 3.87 kPa. Note that measurements were stopped early if the drying capacity of the infra-red gas exchange system was saturated and unable to maintain reduced relative humidity. The intrinsic water use efficiency (_i_WUE) was calculated as _i_WUE = *A*/*g_s_* with *A* being the photosynthetic rate. At every VPD_leaf_ level the steady-state *g_s_*, *A*, E_rate_ (transpiration rate) and _i_WUE after 60 min was calculated. The maximum *g_s_* was calculated as the highest *g_s_* observed across all VPD_leaf_ levels. Segmented regression was performed on the transpiration rate response to increasing VPD_leaf_ for each accession by using a nonlinear mixed effect model in which the intercept was assumed to vary at individual plant level (segmented R package, Muggeo, 2008). This analysis calculates the optimal breakpoint in the transpiration response with a different linear response before and after the breakpoint. To determine the effect of the reduction in stomatal opening on the transpiration, the transpiration reduction (ϕ_E_) was determined according to Franks et al. (1999) and Ryan et al. (2016) (**Figure 1**). For each individual, a linear regression was fitted through the transpiration rate at the first two VPD_leaf_ levels (0.90 and 1.50 kPa). This linear regression was then extrapolated to predict the transpiration rate (E_pred_) at higher VPD_leaf_ levels (2.69, 3.28 and 3.87 kPa) (**Figure 1**). The percentage decrease of the actual measured transpiration rate (E_meas_) compared to E_pred_ (**Figure 1**) was then quantified at each VPD_leaf_ level:

Eq. 1 $${\text{ϕ}}_{E}=1-\frac{E_{meas}}{E_{pred}}$$

The percentage of limitation of the photosynthetic rate (*A*) by *g_s_* reduction was calculated at every VPD_leaf_ level by comparing the measured *A* (*A*_meas_) with the overall maximally measured *A* (*A*_max_) (McAusland et al., 2016):


Eq. 2 $$Limitation\;of\;A=\frac{\sum (A_{max}-A_{meas})}{\sum A_{meas}}$$

Stomatal reduction (ϕ_stom_) with increasing VPD was defined as the absolute slope between stomatal conductance (*g_s_*) and log_e_(VPD_leaf_) as described by Oren et al. (1999):


Eq. 3 $$g_{s}=a-{\text{ϕ}}_{stom}\;\log_{e}VPD_{leaf}$$

where *a* is the estimated *g_s_* at VPD_leaf_ 1 kPa.

### 2.3 Whole-plant transpiration rate

Plants were placed on balances (0.01 g accuracy, Kern, Germany) to register their weight every 10 s. The soil was covered by plastic to avoid evaporation and ensure only water loss through transpiration. Transpiration during each VPD step was calculated by differentiating 5 min average total weight (m*_tot_*) at the start of the VPD level with the 5 min average total weight at the end of the VPD level:


Eq. 4 $$E_{rate}=\frac{\left(m_{tot,t2}-m_{tot,t1}\right)}{LA*\left(t_{2}-t_{1}\right)}$$

Transpiration was normalized by leaf area (LA) and the time (t) passed. LA was quantified by destructive leaf area imaging at the end of the experiment.

Segmented regression was performed on the transpiration rate response to increasing VPD for each accession by using a nonlinear mixed effect model in which the intercept was assumed to vary at plant level. Transpiration reduction (ϕ_E_) was determined according to Eq. 1 with linear regression between the two first VPD levels (0.77 and 1.36 kPa) and comparison between E_pred_ and E_meas_ at the highest level (2.64 kPa).

### 2.4 Statistics

All data processing and statistical analysis were carried out in R (V3.6.2). Genotypic differences were tested by applying analysis of variance (ANOVA) with a *post hoc* Benjamini & Hochberg correction. Significance of the segmented response of transpiration rate to VPD compared to a linear response was determined by the Davies Test (segmented R package, Muggeo, 2008). K-means clustering of accessions was performed on the average scaled output of the segmented regression, the transpiration reduction, the stomatal reduction and photosynthesis limitation, including measurements by leaf gas exchange and by whole-plant transpiration were included (Hartigan and Wong, 1979). Clusters were optimized across 10,000 random sets of cluster centres and plotted on the first two principal components.

## 3 Results

### 3.1 Diverse response to VPD: three phenotypic clusters

**Figure 2:**
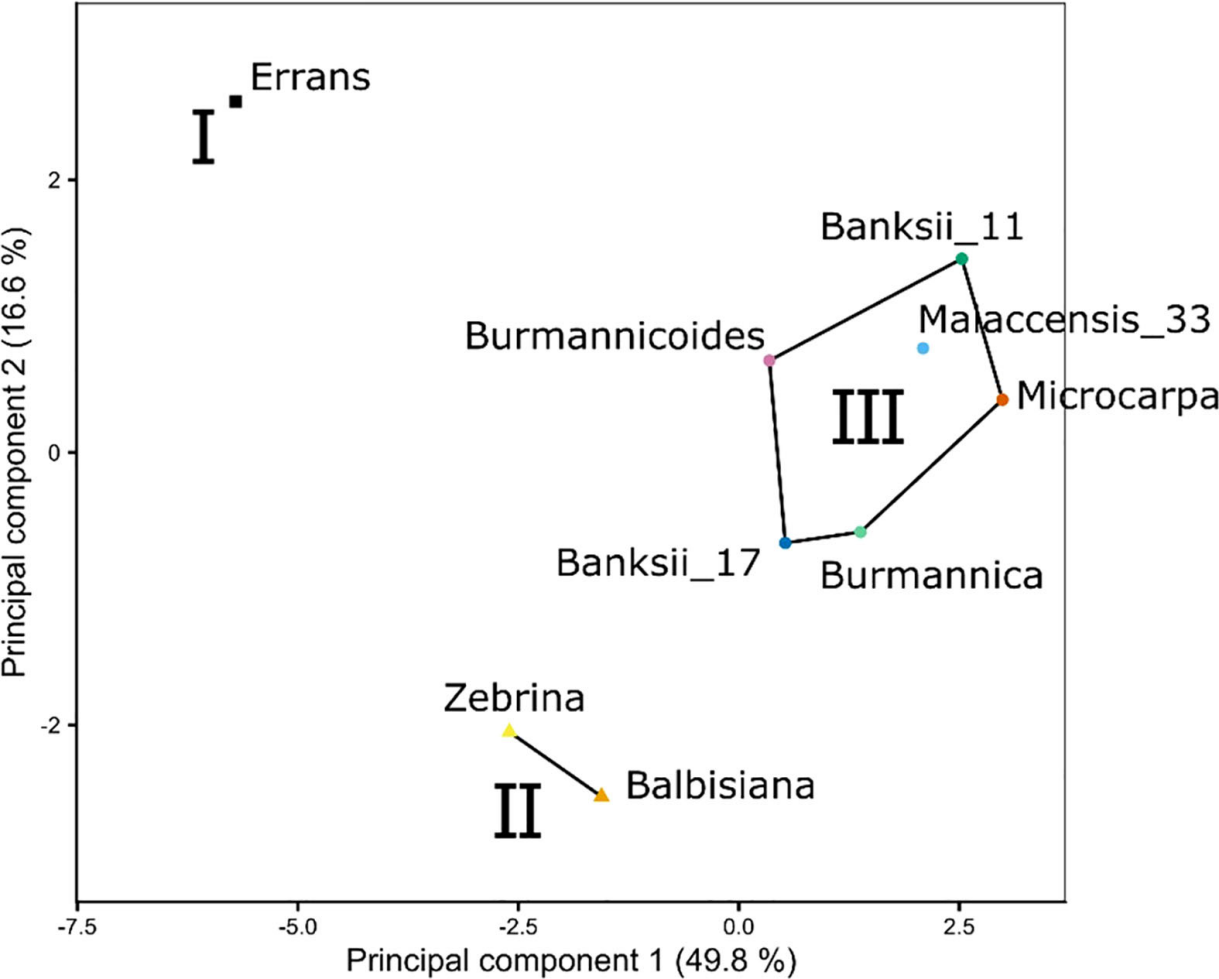
Three phenotypic groups (I, II, III) were defined by k-means clustering based on the stomatal reduction, transpiration reduction and photosynthetic limitation under increasing VPD (see variables in **Table 2**). Both variables measured by leaf gas exchange and whole-plant transpiration were included. Lines and regions represent the three phenotypic groups from k-means clustering plotted along the first two principal components (**Table 2**). The first principal component was mainly determined by the limitation of photosynthetic rate at high VPDs and the transpiration reduction at leaf and whole-plant level. Important variables in the second principal component were the slope before the breakpoint in transpiration rate with increasing VPD and the stomatal reduction.

The transpiration response was measured at leaf and whole-plant level while relative humidity was stepwise decreased and VPDs consequently increased. The response to increasing VPD at leaf and whole-plant level was described by the segmented regression of transpiration rate versus VPD, the transpiration reduction (Eq. 1), the photosynthetic limitation under increasing VPD (Eq. 2) and the stomatal reduction (Eq. 3). K-means clustering was performed on the output variables measured by both leaf gas exchange and whole-plant transpiration (**Table 2**). Three clusters were identified and plotted along the first two principal components (**Figure 2**). The first principal component was mainly determined by the limitation of photosynthetic rate (*A*) at high VPDs and the transpiration reduction at leaf and whole-plant level (**Table 2**). Important variables in the second principal component were the slope before the breakpoint in transpiration rate with increasing VPD and the stomatal reduction (**Table 2**). Cluster I consisted of only one species: *M. acuminata* ssp. *errans* (**Figure 2**). In group II *M. acuminata* ssp. *zebrina* and *M. balbisiana* clustered together (**Figure 2**). Group III contained 6 accessions: *M. acuminata* ssp. *banksii* (2), ssp. *burmannica* (1), ssp. *burmannicoides* (1), ssp. *malaccensis* (1) and ssp. *microcrocarpa* (1) (**Figure 2**).

**Table 2:** Variables included in the k-means clustering and their principal component (PC) loadings.

Variable^1^	Measurement level	PC1 loading^2^	PC2 loading
Limitation of *A* at 3.87 kPa	Leaf gas exchange	-0.35	0.08
Limitation of *A* at 3.28 kPa	Leaf gas exchange	-0.34	0.13
Transpiration reduction at 2.69 kPa	Leaf gas exchange	-0.34	-0.14
Limitation of *A* at 2.69 kPa	Leaf gas exchange	-0.32	0.23
Transpiration reduction at 3.28 kPa	Leaf gas exchange	-0.32	-0.24
Transpiration reduction at 3.87 kPa	Leaf gas exchange	-0.29	-0.34
Transpiration reduction at 2.64 kPa	Whole plant transpiration	-0.29	0.00
Breakpoint in transpiration rate	Leaf gas exchange	0.25	-0.04
Slope after breakpoint in transpiration rate	Whole plant transpiration	0.24	0.19
Limitation of *A* at 2.09 kPa	Leaf gas exchange	-0.22	0.22
Slope after breakpoint in transpiration rate	Leaf gas exchange	0.20	0.28
Slope before breakpoint in transpiration rate	Leaf gas exchange	0.16	-0.39
Breakpoint in transpiration rate	Whole plant transpiration	0.14	0.05
Limitation of *A* at 1.50 kPa	Leaf gas exchange	0.13	-0.03
Slope before breakpoint in transpiration rate	Whole plant transpiration	0.09	-0.49
Stomatal reduction	Leaf gas exchange	0.04	-0.41

^1^Variables measured by leaf gas exchange and whole-plant transpiration were included. ^2^Data were ordered following the absolute value of the first principal component loadings.

### 3.2 Leaf level responses of *g_s_*, transpiration rate and *A* to increasing VPD_leaf_

**Figure 3:**
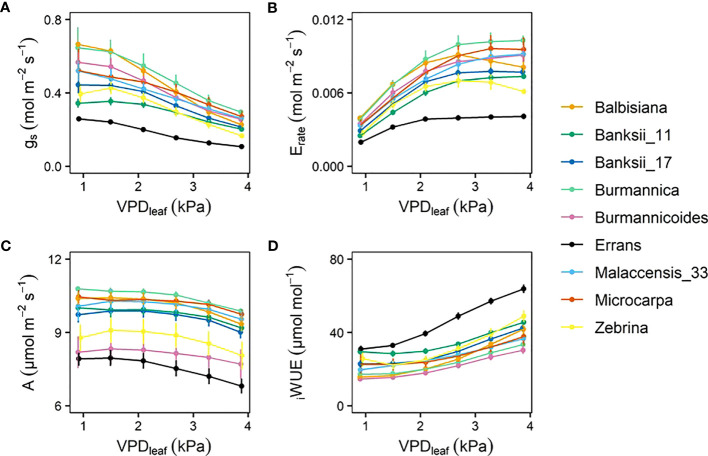
Gas exchange response to step-increases in leaf-to-air vapour pressure deficit (VPD_leaf_) for 9 wild banana accessions. Steady-state response of **(A)** stomatal conductance (g_s_), **(B)** transpiration rate (E_rate_), **(C)** photosynthetic rate **(A)**, **(D)** intrinsic water use efficiency (_i_WUE) to increasing VPD_leaf_. Data represent mean ± se values after 60 min at a specific VPD_leaf_ level (n=3-7). Significance is shown in **Table A.1**.

With increasing VPD_leaf_, *g_s_* decreased in all accessions (**Figure 3A**, **Table A.1**). The transpiration rate initially increased, but eventually reached steady-state or even declined (**Figure 3B**). The transpiration rate and *g_s_* of *M. acuminata* ssp. *errans* were lowest and differed significantly from all other accessions at VPD_leaf_ exceeding 1.50 and 2.09 kPa, respectively (**Figures 3A, B**, **Table A.1**). Under a VPD_leaf_ ≤ 2.9 kPa, the highest transpiration rates and *g_s_* were observed for *M. balbisiana* and *M. acuminata* ssp. *burmannica*. However, when VPD_leaf_ increased further, the *g_s_* of *M. balbisiana* decreased stronger than *M. acuminata* ssp. *burmannica*, translating only in *M. balbisiana* in a lower transpiration rate (**Figures 3A, B**, **Table A.1**). As *g_s_* decreased with increasing VPD_leaf_, the CO_2_ uptake was limited and *A* decreased (**Figure 3C**). The lowest *A* was observed for *M. acuminata* ssp. *errans* and ssp. *burmannicoides*, with significantly lower *A* compared to all other accessions except *M. acuminata* ssp. *zebrina* (**Figure 3C**, **Table A.1**). The intrinsic water use efficiency (_i_WUE) increased with increasing VPD_leaf_ (**Figure 3D**). _i_WUE was highest in *M. acuminata* ssp. *errans* and differed significantly from all other accessions as VPD_leaf_ exceeded 1.5 kPa (**Figure 3D**, **Table A.1**). The lowest _i_WUE were observed for *M. acuminata* ssp. *burmannica* and ssp. *burmannicoides* (**Figure 3D**).

**Figure 4:**
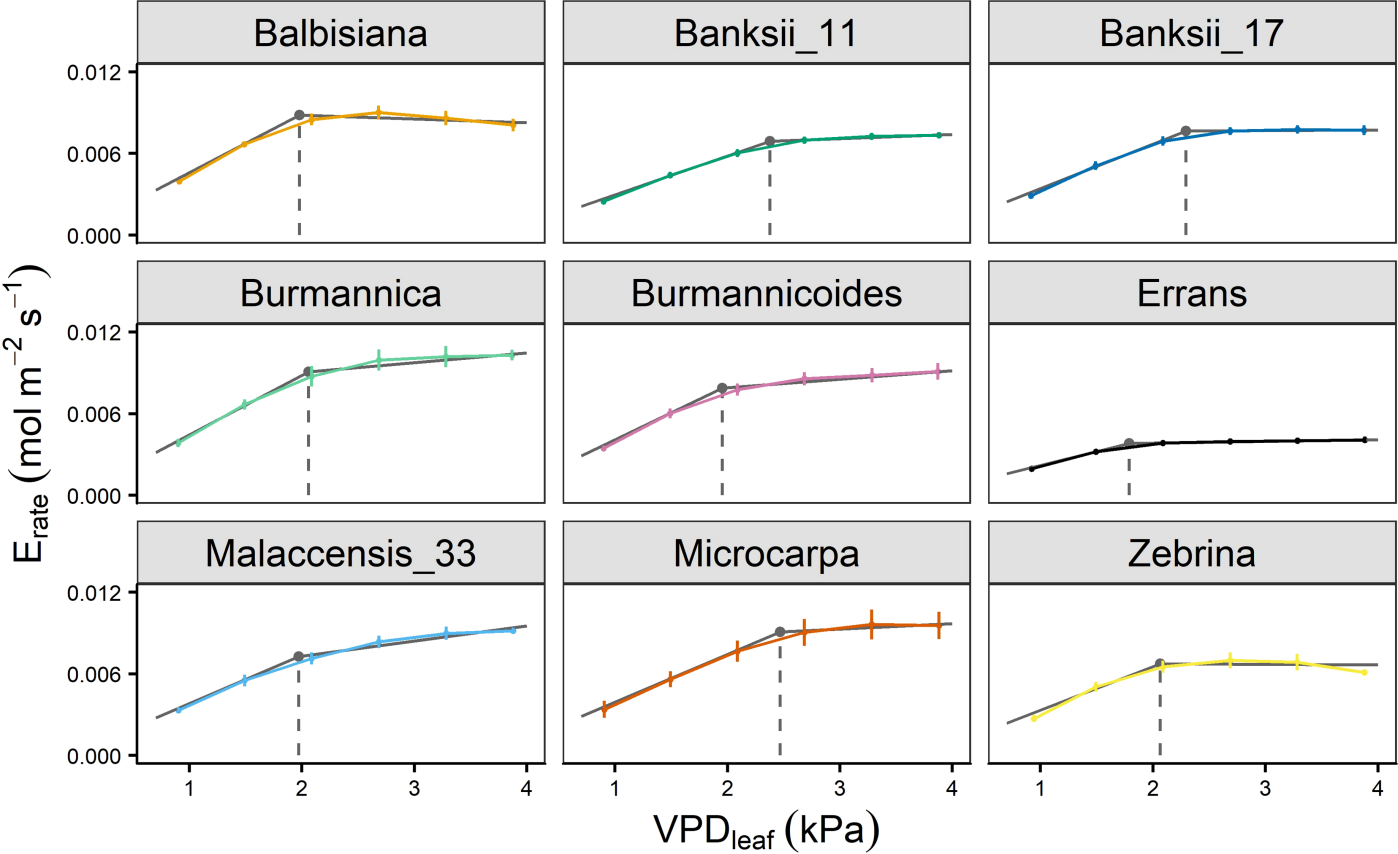
Transpiration rate response of 9 wild banana accessions to step-increases in leaf-to-air vapour pressure deficit (VPD_leaf_). A significant breakpoint in transpiration rate was identified for all accessions (P-value Davies Test < 0.05). Solid grey lines represent slopes of the modelled segmented response. Grey point and dashed grey line represent the breakpoint in transpiration rate and the VPD_leaf_ of the breakpoint. Data represent mean ± se values after 60 min at a specific VPD_leaf_ level (n=3-7).

**Figure 5:**
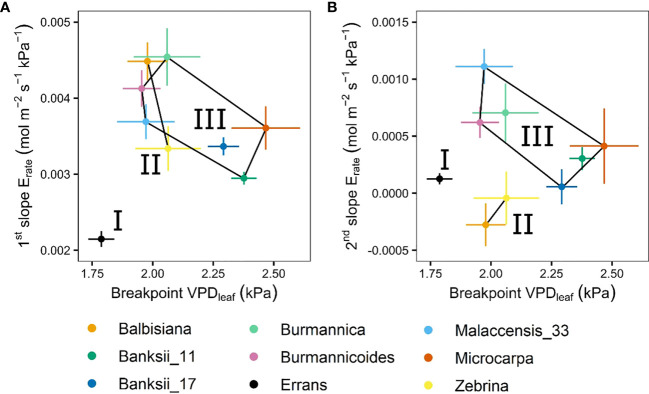
Slopes and breakpoints of the segmented transpiration rate response to step-increases in leaf-to-air vapour pressure deficit (VPD_leaf_). **(A)** Relation between the breakpoint in transpiration rate and the slope before the breakpoint. **(B)** Relation between the breakpoint in transpiration rate and the slope after the breakpoint. Three groups (I, II, III) were defined by k-means clustering and are represented by black lines connecting the included accessions. All segmented responses were significant (P < 0.05). Data represent the optimal estimated value ± se. (n=3-7).

In all accessions there was a decrease in the slope of transpiration rate versus VPD_leaf_ (**Figure 3B**). This response was described by a segmented regression with a specified breakpoint after which the slope of the transpiration rate decreases. A significant breakpoint in transpiration rate in response to VPD_leaf_ was identified in all accessions (**Figure 4**). Across accessions the breakpoints ranged between 1.75 and 2.5 kPa with *M. acuminata* ssp. *errans* having a significant breakpoint at the lowest VPD_leaf_ (**Figures 4**, **5**). Two *M. acuminata* ssp. *banksii* accessions and ssp. *microcarpa* showed the highest breakpoint in transpiration rate (**Figures 4**, **5**). The groups defined by k-means clustering differed in their segmented transpiration response (**Figure 5**). Group I consisted only of *M. acuminata* ssp. *errans*, the subspecies with a breakpoint (a reduction in transpiration rate) at the lowest VPD_leaf_, as well as the lowest slope (the lowest E_rate_) before the breakpoint (**Figure 5**). Group II, consisting of *M. acuminata* ssp. *zebrina* and *M. balbisiana*, had a breakpoint at a relatively low VPD_leaf_ around 2 kPa and a negative slope after the breakpoint (**Figure 5**). This negative slope indicates a net decrease in transpiration rate, which was not observed in the other accessions. In group III all accessions kept relatively high transpiration rates at relatively high VPD_leaf_. *Musa acuminata* ssp. *burmannica*, ssp. *burmannicoides* and ssp. *malaccensis* had a breakpoint at relatively low VPD_leaf_, but maintained a high slope of transpiration rate afterwards while the *M. acuminata* ssp. *banksii* accessions and ssp. *microcarpa* showed only a significant breakpoint in transpiration rate at higher VPD_leaf_, (**Figure 5**).

**Figure 6:**
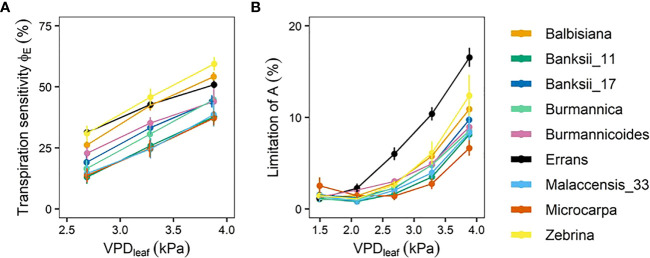
Transpiration reduction (ϕ_E_) and limitation of photosynthetic rate **(A)** with increasing leaf-to-air vapour pressure deficit (VPD_leaf_). **(A)** ϕ_E_ in response to increasing VPD_leaf_. ϕ_E_ was determined as shown in Eq. 1. **(B)** Limitation of A in response to increasing VPD_leaf_. The limitation of A was determined as shown in Eq. 2. Data represent mean ± se. (n=3-7). Significance is shown in **Tables A.2**, **A.3**.

The transpiration reduction (ϕ_E_) (Eq. 1, **Figure 1**) representing the increase in stomatal resistance with increasing VPD_leaf_ also differed significantly across accessions (**Figure 6A**, **Table A.2**). Reductions in transpiration ranged between 37 and 59% at the highest VPD_leaf_ of 3.87 kPa (**Figure 6A**, **Table A.2**). The highest reductions in transpiration were observed for *M. acuminata* ssp. *errans*, ssp. *zebrina* and *M. balbisiana* (**Figure 6A**). The transpiration reduction of group I and II was significantly higher compared to group III at all VPD_leaf_ levels (**Figure 6A**, **Table A.2**).

The decrease in stomatal opening with increasing VPD_leaf_ limited the photosynthetic rate (*A*). In all accessions there was a significant increase in the limitation of *A* with increasing VPD_leaf_ (P < 0.01) and the limitation ranged from 7 to 17% at the highest VPD_leaf_ level (**Figure 6B**, **Table A.3**). The limitation of *A* was highest in *M. acuminata* ssp. *errans* from VPD_leaf_ 2.69 kPa onwards, followed by *M. acuminata* ssp. *zebrina* and *M. balbisiana* (**Figure 6B**, **Table A.3**). The limitation of *A* was significantly higher in group I compared to group II and III from VPD_leaf_ 2.69 kPa onwards (**Table A.3**). At VPD_leaf_ of 3.28 and 3.87 kPa group II had a significantly higher *A* limitation compared to group III (**Table A.3**). Across accessions the limitation of *A* at higher VPD_leaf_ (≥ 2.69 kPa) was significantly correlated to the breakpoint in transpiration rate (R² = 0.47-0.57; **Figure A.1**). Similarly, the limitation of *A* and the transpiration reduction at higher VPD_leaf_ (≥ 2.69 kPa) were significantly correlated (R² = 0.53-0.58; **Figure A.1**). These correlations indicate that strong reductions in transpiration at high VPD_leaf_ result in higher *A* limitations.

**Figure 7:**
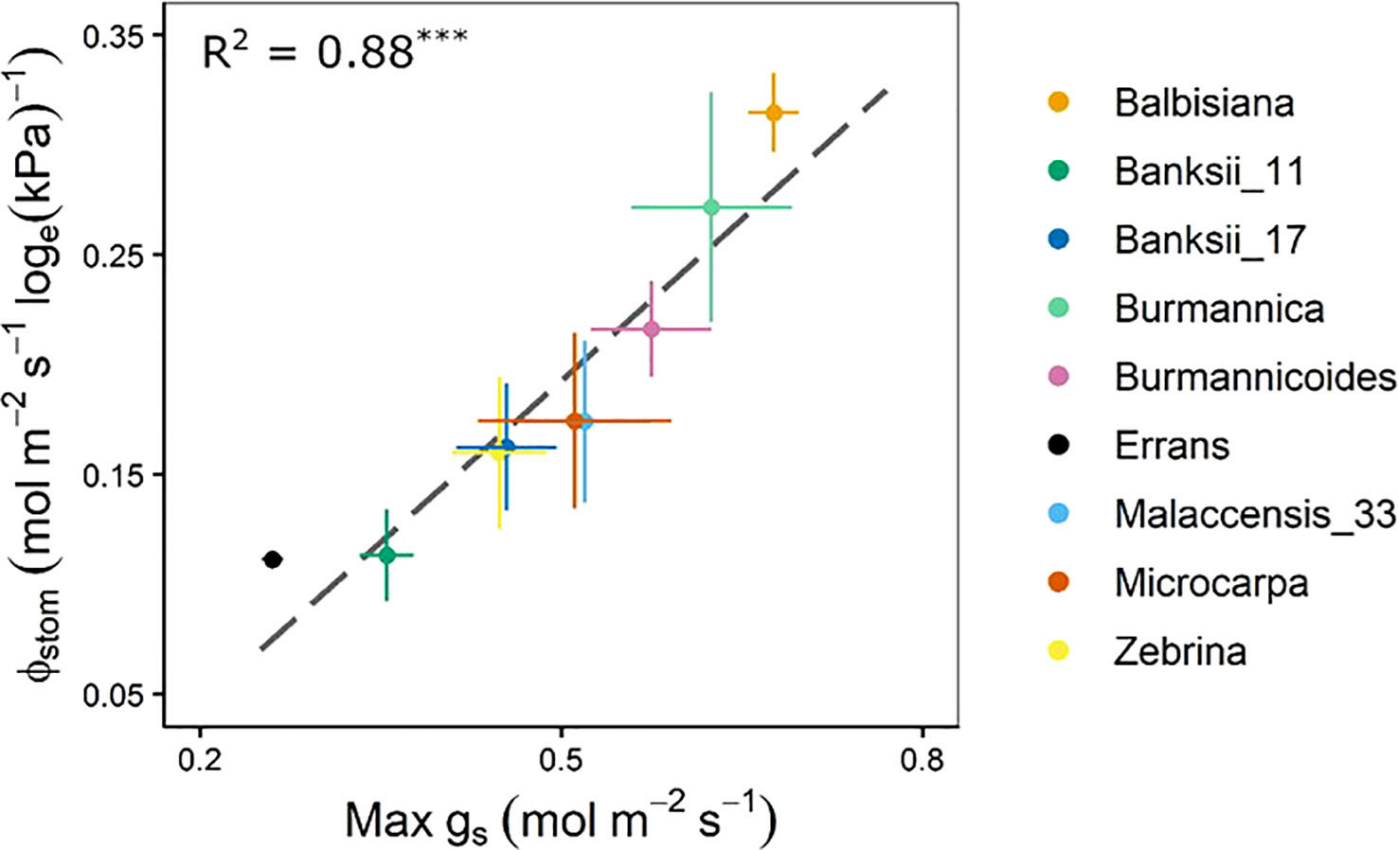
Stomatal reduction (ϕ_stom_) in relation to the maximum observed stomatal conductance (max g_s_). The ϕ_stom_ and max g_s_ were significantly correlated (R² = 0.88, P < 0.001). Data represent mean ± se (n=3-7). Significance is shown in **Table A.4**.

The stomatal reduction (ϕ_stom_), defined as the slope of *g_s_* versus log_e_(VPD_leaf_) (Eq. 3) differed significantly across accessions (**Table A.4**). Highest stomatal reduction was observed in *M. balbisiana*, while *M. acuminata* ssp. *errans* showed lowest reduction (**Figure 7**, **Table A.4**). The stomatal reduction was strongly correlated to the maximum observed *g_s_* (R² = 0.88, **Figures 7**, **A.1**). No significant differences across previously described groups was observed (**Table A.4**).

### 3.3 Whole-plant transpiration rate responses corroborate leaf measurements

**Figure 8:**
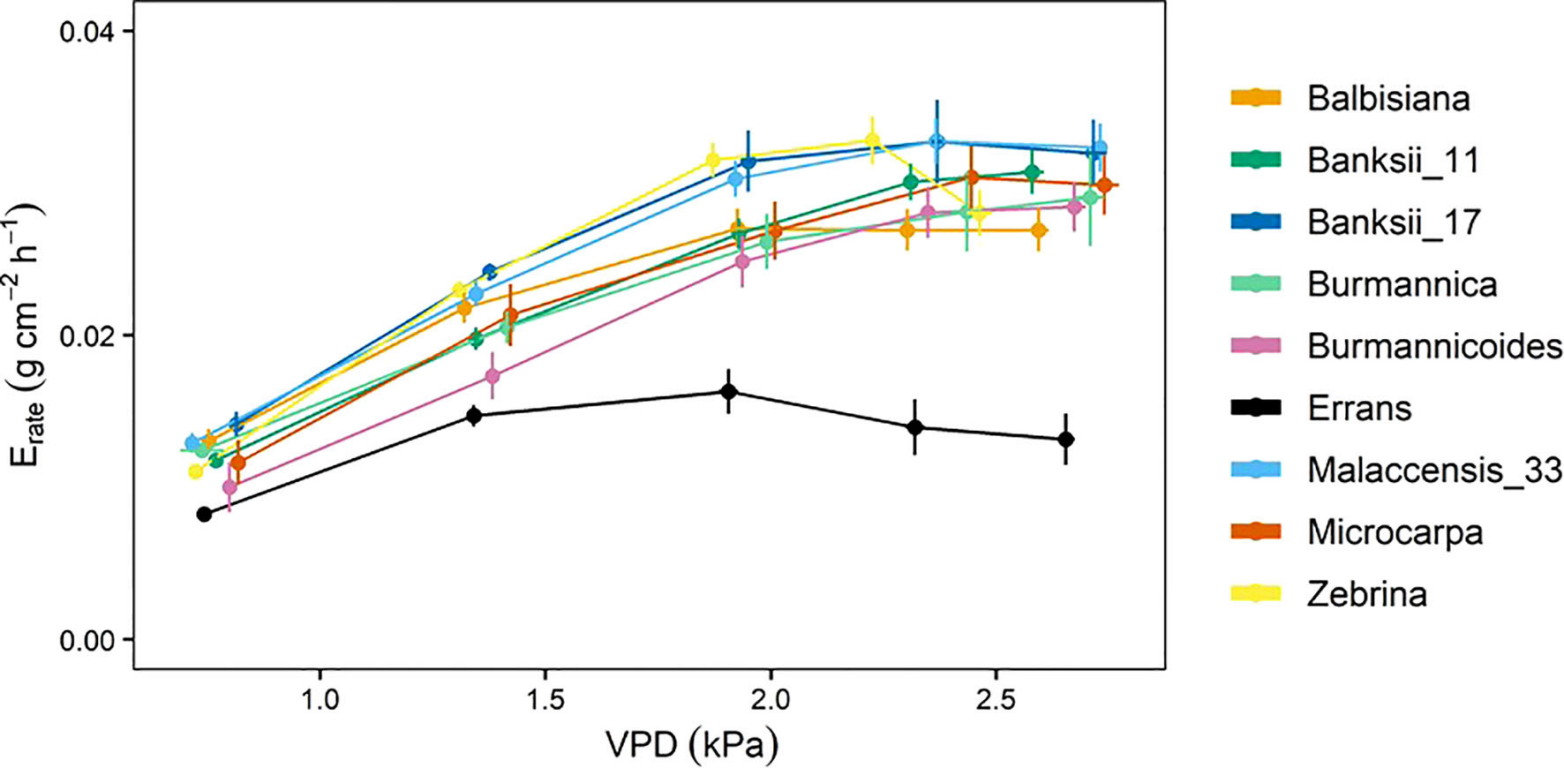
Whole-plant transpiration rate (E_rate_) response to step-increases in air vapour pressure deficit (VPD) for 9 wild banana accessions. Note that VPD values slightly differed between accessions depending on the maximal drying capacity of the growth chamber. Data represent mean ± se values after 60 min at a specific VPD level (n=4-8). Significance is shown in **Table A.5**.

The whole-plant transpiration rate increased between 98 and 197% with increasing VPD (**Figure 8**). The lowest transpiration rates were observed for *M. acuminata* ssp. *errans* with significant differences compared to all other accessions from VPD 1.93 kPa and beyond (**Figure 8**, **Table A.5**). Transpiration rates of all other accessions were double compared to *M. acuminata* ssp. *errans* at the highest VPD level (**Figure 8**, **Table A.5**).

**Figure 9:**
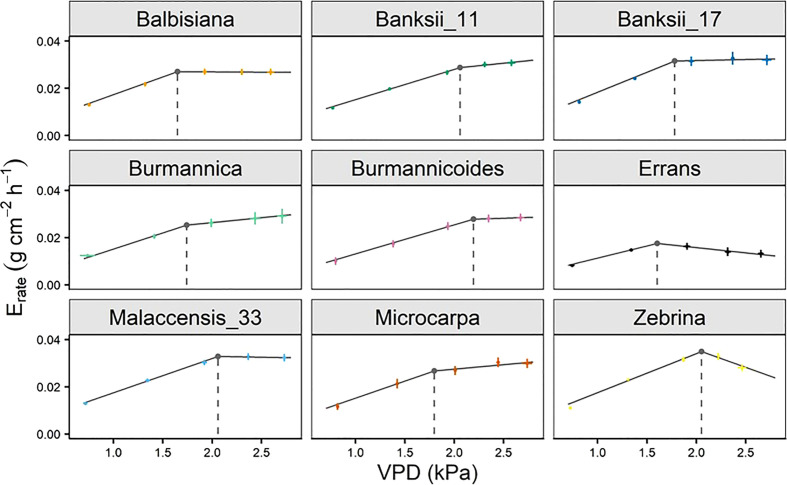
Whole-plant transpiration rate (E_rate_) response of 9 wild banana accessions to step-increases in air vapour pressure deficit (VPD). A significant breakpoint in transpiration rate was identified for all accessions (P-value Davies Test < 0.05). Solid grey lines represent slopes of the modelled segmented response. Grey point and dashed grey line represent the breakpoint in transpiration rate and the VPD of the breakpoint. Data represent mean ± se (n=4-8).

**Figure 10:**
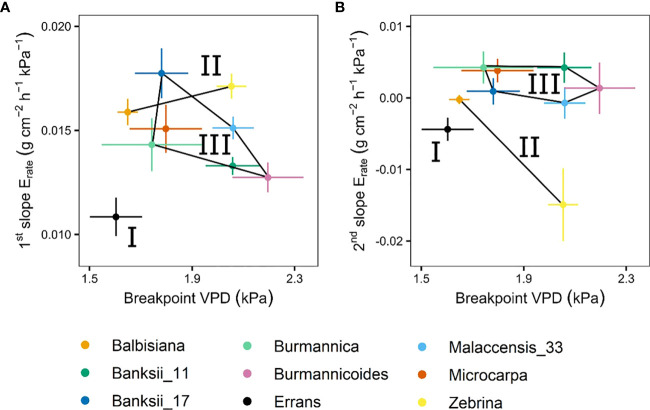
Slopes and breakpoints of the segmented whole-plant transpiration rate (E_rate_) response to step-increases in air vapour pressure deficit (VPD). **(A)** Relation between the breakpoint in whole-plant transpiration rate and the slope before the breakpoint. **(B)** Relation between the breakpoint in whole-plant transpiration rate and the slope after the breakpoint. Three groups (I, II, III) were defined by k-means clustering and are represented by black lines connecting the included accessions. All segmented responses were significant (P < 0.05). Data represent the optimal estimated value ± se (n = 4-8).

A significant breakpoint in whole-plant transpiration rate response to VPD was identified in all accessions (**Figure 9**). The breakpoints ranged between 1.6 and 2.2 kPa, with *M. acuminata* ssp. *errans* and *M. balbisiana* having the lowest breakpoint (**Figures 9**, **10**). The slope after the breakpoint was strongly negative in *M. acuminata* ssp. *errans* and ssp. *zebrina* (**Figures 9**, **10**). Accessions belonging to group I or II thus showed breakpoints in transpiration rate at lower VPD values and/or strongly negative second slopes (**Figure 10**).

**Figure 11:**
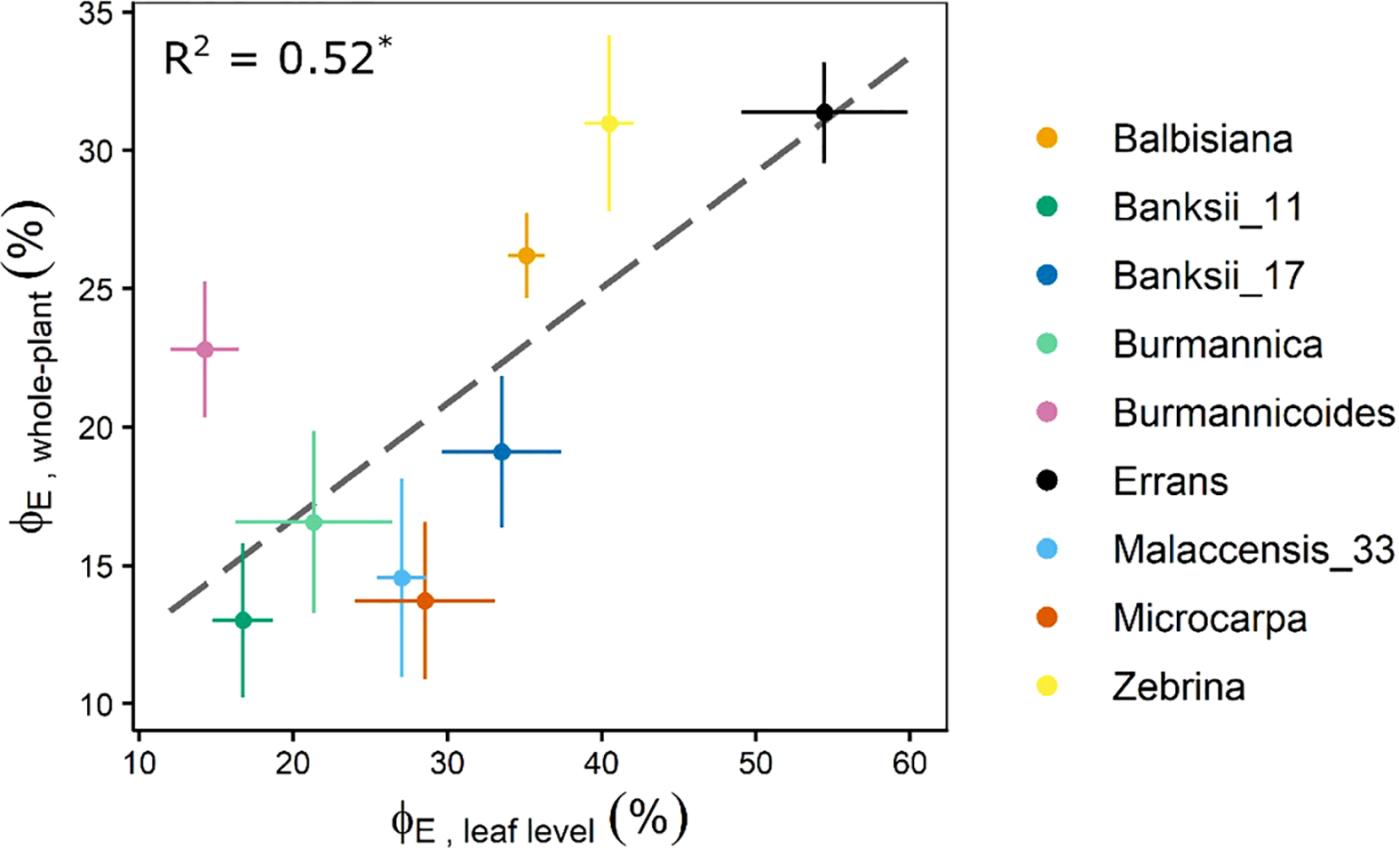
Transpiration reduction measured at whole-plant level (ϕ_E_, whole-plant) at VPD 2.64 kPa in relation to the transpiration reduction measured at leaf level (ϕ_E_, leaf level) at VPDleaf 2.69 kPa. The ϕ_E_ at leaf and whole-plant level were significantly correlated (R² = 0.52, P < 0.05). Data represent mean ± se (n=4-8). Significant differences between accessions or groups are indicated in **Tables A.2**, **A.6**.

The whole-plant transpiration reduction (ϕ_E_) (Eq. 1, **Figure 1**) of *M. acuminata* ssp. *errans* was significantly higher compared to all other accessions (**Figure 11**, **Table A.6**). The second highest transpiration reduction was observed for *M. acuminata* ssp*. zebrina* and *M. balbisiana* (**Figure 11**, **Table A.6**). Group I (*M. acuminata* ssp. *errans*) showed a significantly higher transpiration reduction compared to group II and III (**Table A.6**). Group II (*M. acuminata* ssp*. zebrina* and *M. balbisiana*) showed a significantly higher transpiration reduction compared to group III (*Musa acuminata* ssp. *burmannica*, ssp. *burmannicoides*, ssp. *malaccensis*, ssp. *banksii* and ssp. *microcarpa*) (**Table A.6**).

The whole-plant transpiration reduction was significantly correlated to the transpiration reduction measured at leaf level at similar VPD (R² = 0.52, **Figures 11**, **A.1**). Similarly, the whole-plant transpiration reduction was significantly correlated to the limitation of *A* measured at leaf level for VPD_leaf_ exceeding 2.1 kPa (R² = 0.50 - 0.73, **Figure A.1**).

## 4 Discussion

Diversity in transpiration patterns with increasing VPD has been observed among different genotypes of many crops such as maize, sorghum and soybean (Fletcher et al., 2007; Gholipoor et al., 2010; Yang et al., 2012). We observed a significant change in the transpiration rate of 9 wild banana gene bank accessions already at VPD levels between 1.6 and 2.5 kPa (**Figures 4**, **9**). These values are in line with the general transpiration rate reduction of banana at VPD 2 to 2.3 kPa reported by Carr (2009) and the modelled VPD responses of Eyland et al. (2022). The breakpoints in transpiration rate were at similar VPDs compared to other crops (Gholipoor et al., 2010; Yang et al., 2012; Ryan et al., 2016). However, in other crops several genotypes were identified without a breakpoint as they maintained a linear increase in transpiration rate with increasing VPD (Fletcher et al., 2007; Gholipoor et al., 2010; Yang et al., 2012). Moreover, temperature and other environmental factors like radiation and soil water potential have been shown to interact with VPD in banana (Eyland et al., 2022). These complex interactions explain why a fixed VPD level per accession, where a reduction in transpiration takes place, cannot be defined without taking the other environmental conditions in account.

The wild banana accessions clustered in three groups based on their leaf gas exchange and whole-plant transpiration response to VPD (**Figure 2**). Accessions of group I and II, *M. acuminata* ssp. *errans*, *M. acuminata* ssp. *zebrina* and *M. balbisiana*, showed the highest transpiration rate limitations. This is in line with our previous observations under fluctuating conditions: *M. balbisiana* showed together with *M. acuminata ssp. errans* the most pronounced response by strongly decreasing their transpiration rate (Eyland et al., 2022). As reported by Oren et al. (1999), the stomatal reduction was significantly correlated to the maximum g*_s_* (**Figures 7**, **A.1**). This indicates that accessions with higher g_s_ under low VPD_leaf_ show higher stomatal closure at increasing VPD_leaf_. However, *M. acuminata* ssp. *errans* (group I) showed a very strong stomatal response, despite its low g*_s_*. As a consequence of this strong stomatal restriction, the _i_WUE of *M. acuminata* ssp. *errans* was significantly higher compared to all other accessions (**Figure 3D**). In contrast to the very conservative behaviour of *M. acuminata* ssp. *errans*, the accessions of group II displayed high *g_s_* and *A* when VPD_leaf_ was favourable in addition to early or strong transpiration rate reductions at high VPD_leaf_. This behaviour is assumed to be beneficial in drought-prone areas with periods of high VPD (Sadok and Sinclair, 2010; Vadez, 2014), as water is used efficiently and saved for later in the growing season. Some accessions of group III also showed a breakpoint in transpiration at a relatively low VPD_leaf_, but a high transpiration rate was kept and a net transpiration increase continued with rising VPD_leaf_ (**Figures 4**, **5**). Hence, these accessions display a more risk taking behaviour, thereby risking hydraulic failure (Sade et al., 2012).

Transpiration reductions at leaf level were validated at the whole-plant level (**Figure 11**). Accessions belonging to group I and II showing the highest transpiration rate limitations at leaf level also showed significant breakpoints in whole-plant transpiration rates. These breakpoints occurred at low VPDs after which transpiration rate increases were limited (**Figures 5**, **9**).

The physiological and molecular origin of the observed genotypic variability still remains to be elucidated. This evaluation of leaf and whole-plant responses gives an idea on how fast imbalances in water supply and demand develop, as well as on the stomatal responsiveness to these imbalances. The restricted transpiration phenotype under high VPD has been linked in other crops to a limited hydraulic conductance by reduced expression of specific aquaporins (Sadok and Sinclair, 2010; Devi et al., 2015; Reddy et al., 2017). However, the high correlation between hydraulic and stomatal conductance and the challenge to measure hydraulic conductance in banana makes it challenging to separate these two processes in the current experimental setup (Turner et al., 2007). Nevertheless, given the low modelled maximal transpiration rate of *M. acuminata* ssp. *errans* (Eyland et al., 2022) and the low observed constituent conductance and transpiration (**Figures 3A, B**) it can be hypothesised that this species is characterised by a low hydraulic conductance. In contrast, group II and III accessions are hypothesized to have a better hydraulic conductance, allowing increased transpiration with increased evaporative demand. However, the early and strong interference of group II accessions, i.e. *M. acuminata* ssp. *zebrina* and *M. balbisiana*, might point towards a high hydraulic capacity but a fast stomatal reaction to an increasing water imbalance. The robustness of *M. balbisiana* to increased evaporative demand has already been reported in literature. During a collection mission in Papua New Guinea, it was the only species found almost always in open habitats (Eyland et al., 2020). Under these conditions of high evaporative demands, survival would only be possible if the species is equipped with a high water uptake and transport capacity, as well as reduced stomatal conductance (Eyland et al., 2020). Genotypic variability in stomatal response might be dependent on the speed of ABA anabolism and catabolism, as well as on the number of ABA receptors. Additionally physiological and molecular measurements are required to validate these hypotheses.

As demonstrated in other crops, identification of this conservative behaviour towards VPD, opens up possibilities to improve drought tolerance of cultivated banana hybrids. *M. balbisiana* is a parent to many edible bananas belonging to the AAB, ABB and AB genome groups and their subgroups. Moreover, in line with the conservative behaviour of *M. balbisiana* in response to VPD (**Figures 3**, **4**, **6**, **8**), it has been indicated in many studies that edible bananas with a high portion of B genes are related to drought tolerance (Ekanayake et al., 1994; Thomas et al., 1998; Turner and Thomas, 1998; Thomas and Turner, 2001; Vanhove et al., 2012; Kissel et al., 2015; Van Wesemael et al., 2018; van Wesemael et al., 2019; Eyland et al., 2021; Uwimana et al., 2021; Eyland et al., 2022). Also *M. acuminata* ssp. *zebrina* is a parent to several edible bananas (Carreel et al., 2002; Perrier et al., 2011; Němečková et al., 2018; Baurens et al., 2019; Martin et al., 2020a; Martin et al., 2020b; Jeensae et al., 2021), among others the East-African highland banana subgroup (i.e. Mutika/Lujugira). The East-African highland banana subgroup, endemic to the East-African highlands, is due to its risk taking behaviour sensitive to drought (Kissel et al., 2015; van Wesemael et al., 2019; Eyland et al., 2021; Uwimana et al., 2021). Hence, identification of drought tolerance traits in *M. acuminata* ssp. *zebrina* populations provides opportunities to mitigate climate change impacts in this and all other important subgroups. So far, not much is known about the contribution of *M. acuminata* ssp. *errans* to edible bananas. The accession screened in this study and representing *M. acuminata* ssp. *errans*, has been proved to be complex in genome with ancestries coming from ‘*malaccensis*’, ‘*zebrina*’ and ‘*burmannica/siamea*’ (Martin et al., 2020b).

## 5 Conclusions

The reduction of transpiration response to high VPD is a key trait for water use efficiency and diversity among wild banana relatives was observed. Reductions in transpiration ranging between 37 and 59%, translated in an increased WUE of 54 to 166%. *M. acuminata* ssp. *errans*, on the one hand, responded most conservative, but was also characterized by low g*_s_* overall. *M. acuminata* ssp*. zebrina* and *M. balbisiana*, on the other hand, showed strong stomatal closure while maintaining relatively high carbon uptake under low VPD. These two (sub)species thus show favourable responses for a specific sub-trait linked to high water use efficiency, providing a potential basis for the identification of parent material for breeding more drought resilient bananas.

## Data availability statement

The raw data supporting the conclusions of this article will be made available by the authors, without undue reservation.

## Author contributions

SC and RS wrote the concepts for funding. DE performed the experiments and analyzed the data. SC supervised the experiments. SC, CG and DE wrote the manuscript. All authors contributed to the article and approved the submitted version.
