# Supplementary material for: Unravelling the diversity in water usage among wild banana species in response to vapour pressure deficit
Source: Front Plant Sci. 2023 Aug 21;14:1068191. doi: 10.3389/fpls.2023.1068191 (PMC10475999; doi:10.3389/fpls.2023.1068191)
Supplement: Supplementary file 1 [file DataSheet_1.docx]

# Appendix

**Table A.1:** Steady-state response of stomatal conductance (*g_s_*), transpiration rate (E_rate_), photosynthetic rate (*A*) and intrinsic water use efficiency (_i_WUE) to step-increases in leaf-to-air vapour pressure deficit (VPD_leaf_). Data represent mean±se after 60 min at a VPD_leaf_ level. VPD_leaf_ represents the mean at each level over all measurements. Different letters indicate significant differences between genotypes (P<0.05; A>B>C>D>E>F).

| **genotype** | **VPD_leaf_ 0.91 kPa** | **VPD_leaf_ 1.50 kPa** | **VPD_leaf_ 2.09 kPa** | **VPD_leaf_ 2.69 kPa** | **VPD_leaf_ 3.28 kPa** | **VPD_leaf_ 3.87 kPa** | ***n*** |
| --- | --- | --- | --- | --- | --- | --- | --- |
| ***g_s_ (mol m^-2^s^-1^)*** | | | | | | | |
| Errans | 0.26±0.01 (E) | 0.24±0.01 (E) | 0.20±0.01 (D) | 0.15±0.01 (D) | 0.13±0.01 (E) | 0.11±0.01 (F) | 5 |
| Balbisiana | 0.66±0.02 (A) | 0.63±0.02 (A) | 0.52±0.03 (A) | 0.41±0.02 (AB) | 0.30±0.02 (ABCD) | 0.23±0.02 (BCD) | 5 |
| Zebrina | 0.4±0.06 (CDE) | 0.43±0.03 (CD) | 0.37±0.03 (BC) | 0.30±0.03 (C) | 0.23±0.02 (D) | 0.17±0.01 (E) | 7 |
| Banksii_11 | 0.34±0.02 (DE) | 0.35±0.02 (DE) | 0.34±0.02 (C) | 0.29±0.01 (C) | 0.24±0.01 (CD) | 0.20±0.01 (DE) | 5 |
| Banksii_17 | 0.44±0.05 (BCD) | 0.44±0.04 (CD) | 0.41±0.03 (BC) | 0.33±0.01 (BC) | 0.26±0.01 (BCD) | 0.21±0.01 (CD) | 6 |
| Burmannica | 0.65±0.11 (AB) | 0.62±0.07 (AB) | 0.55±0.07 (A) | 0.45±0.05 (A) | 0.36±0.03 (A) | 0.30±0.01 (A) | 4 |
| Burmannicoides | 0.57±0.06 (ABC) | 0.54±0.05 (ABC) | 0.47±0.04 (AB) | 0.37±0.03 (ABC) | 0.30±0.02 (ABC) | 0.26±0.02 (ABC) | 3 |
| Malaccensis_33 | 0.52±0.06 (ABCD) | 0.48±0.05 (BCD) | 0.42±0.04 (ABC) | 0.37±0.03 (ABC) | 0.31±0.02 (ABC) | 0.26±0.01 (ABC) | 3 |
| Microcarpa | 0.52±0.12 (ABCD) | 0.49±0.06 (BCD) | 0.46±0.06 (AB) | 0.4±0.05 (AB) | 0.34±0.04 (AB) | 0.27±0.03 (AB) | 4 |
| ***E_rate_ (mol m^-2^s^-1^)*** | | | | | | | |
| Errans | 0.0020  ±0.0001 (E) | 0.0032  ±0.0001 (D) | 0.0039  ±0.0002 (D) | 0.0040  ±0.0002 (D) | 0.0040  ±0.0002 (E) | 0.0041  ±0.0002 (F) | 5 |
| Balbisiana | 0.0040  ±0.0001 (A) | 0.0067  ±0.0001 (A) | 0.0085  ±0.0003 (A) | 0.0091  ±0.0004 (AB) | 0.0086  ±0.0005 (ABCD) | 0.0081  ±0.0005 (BCD) | 5 |
| Zebrina | 0.0027  ±0.0003 (CDE) | 0.0050  ±0.0004 (BC) | 0.0065  ±0.0005 (BC) | 0.007  ±0.0006 (C) | 0.0069  ±0.0006 (D) | 0.0061  ±0.0002 (E) | 7 |
| Banksii_11 | 0.0025  ±0.0002 (DE) | 0.0044  ±0.0002 (C) | 0.0060  ±0.0003 (C) | 0.0070  ±0.0003 (C) | 0.0073  ±0.0003 (CD) | 0.0073  ±0.0002 (DE) | 5 |
| Banksii_17 | 0.0029  ±0.0002 (BCD) | 0.0051  ±0.0003 (BC) | 0.0069  ±0.0003 (BC) | 0.0076  ±0.0003 (BC) | 0.0078  ±0.0003 (BCD) | 0.0077  ±0.0004 (CD) | 6 |
| Burmannica | 0.0038  ±0.0003 (AB) | 0.0067  ±0.0004 (A) | 0.0087  ±0.0007 (A) | 0.0099  ±0.0008 (A) | 0.0102  ±0.0008 (A) | 0.0103  ±0.0004 (A) | 4 |
| Burmannicoides | 0.0034  ±0.0002 (ABC) | 0.0060  ±0.0004 (AB) | 0.0078  ±0.0004 (AB) | 0.0086  ±0.0005 (ABC) | 0.0088  ±0.0005 (ABC) | 0.0091  ±0.0006 (ABC) | 3 |
| Malaccensis_33 | 0.0033  ±0.0002 (ABCD) | 0.0055  ±0.0004 (ABC) | 0.0071  ±0.0004 (ABC) | 0.0083  ±0.0005 (ABC) | 0.009  ±0.0005 (ABC) | 0.0092  ±0.0002 (ABC) | 3 |
| Microcarpa | 0.0034  ±0.0006 (ABCD) | 0.0056  ±0.0006 (ABC) | 0.0077  ±0.0008 (AB) | 0.009  ±0.0010 (AB) | 0.0096  ±0.0011 (AB) | 0.0095  ±0.0010 (AB) | 4 |
| ***A (µmol m^-2^s^-1^)*** | | | | | | | |
| Errans | 7.92±0.29 (C) | 7.96±0.33 (C) | 7.84±0.32 (C) | 7.52±0.32 (D) | 7.2±0.32 (D) | 6.81±0.31 (D) | 5 |
| Balbisiana | 10.39±0.25 (A) | 10.41±0.26 (A) | 10.37±0.25 (A) | 10.21±0.27 (A) | 9.84±0.33 (A) | 9.34±0.31 (AB) | 5 |
| Zebrina | 8.78±0.55 (BC) | 9.09±0.53 (BC) | 9.04±0.52 (BC) | 8.88±0.51 (BC) | 8.55±0.5 (BC) | 8.06±0.58 (BC) | 7 |
| Banksii_11 | 10.01±0.23 (AB) | 9.92±0.28 (AB) | 9.94±0.24 (AB) | 9.82±0.22 (AB) | 9.63±0.2 (AB) | 9.18±0.19 (AB) | 5 |
| Banksii_17 | 9.73±0.33 (AB) | 9.88±0.29 (AB) | 9.88±0.28 (AB) | 9.74±0.27 (AB) | 9.5±0.26 (AB) | 9.01±0.26 (ABC) | 6 |
| Burmannica | 10.79±0.08 (A) | 10.69±0.15 (A) | 10.66±0.15 (A) | 10.54±0.15 (A) | 10.2±0.13 (A) | 9.87±0.09 (A) | 4 |
| Burmannicoides | 8.19±0.65 (C) | 8.33±0.65 (C) | 8.28±0.64 (C) | 8.14±0.61 (CD) | 7.98±0.6 (CD) | 7.7±0.61 (CD) | 3 |
| Malaccensis_33 | 10.07±0.59 (AB) | 10.28±0.49 (AB) | 10.26±0.44 (AB) | 10.15±0.39 (AB) | 9.95±0.36 (AB) | 9.54±0.36 (A) | 3 |
| Microcarpa | 10.46±0.27 (A) | 10.31±0.26 (AB) | 10.35±0.26 (AB) | 10.29±0.23 (A) | 10.15±0.19 (A) | 9.75±0.19 (A) | 4 |
| ***iWUE (µmol mol^-1^)*** | | | | | | | |
| Errans | 30.99±1.84 (A) | 32.98±1.09 (A) | 39.37±1.69 (A) | 48.89±2.03 (A) | 57.14±1.89 (A) | 63.78±2.39 (A) | 5 |
| Balbisiana | 15.66±0.35 (B) | 16.61±0.39 (DE) | 20.15±1.1 (CD) | 25.38±1.04 (CD) | 33.6±1.62 (BCD) | 41.8±2.44 (BCD) | 5 |
| Zebrina | 25.65±4.33 (AB) | 22.22±2.28 (CD) | 25±1.96 (BC) | 31.13±2.42 (BC) | 39.13±3.15 (B) | 48.88±3.3 (B) | 7 |
| Banksii_11 | 29.47±1.51 (A) | 28.45±1.77 (AB) | 29.84±1.62 (B) | 33.62±1.14 (B) | 39.94±1.02 (B) | 45.55±1.34 (BC) | 5 |
| Banksii_17 | 22.81±1.84 (AB) | 23.02±1.62 (BC) | 24.68±1.4 (BC) | 29.71±1.25 (BC) | 36.5±1.53 (BC) | 42.31±1.77 (BCD) | 6 |
| Burmannica | 17.21±2.88 (AB) | 17.46±1.54 (CDE) | 19.99±2.11 (CD) | 23.67±2.13 (CD) | 28.9±2.37 (CD) | 33.49±1.26 (DE) | 4 |
| Burmannicoides | 14.55±0.76 (B) | 15.57±1.38 (E) | 17.94±1.37 (D) | 21.9±1.54 (D) | 26.6±1.87 (D) | 30.31±2.11 (E) | 3 |
| Malaccensis_33 | 19.63±1.23 (AB) | 21.95±1.66 (BCDE) | 24.65±1.36 (BCD) | 27.77±1.17 (BCD) | 32.07±1.42 (BCD) | 36.61±0.95 (CDE) | 3 |
| Microcarpa | 22.55±5.61 (AB) | 22.47±3.36 (BCDE) | 23.73±3.4 (BCD) | 27.05±4.23 (BCD) | 32.06±4.89 (BCD) | 37.68±4.9 (CDE) | 4 |

**Table A.2:** Transpiration reduction (ɸ_E_) of leaf gas exchange measurements at three different leaf-to-air vapour pressure deficits (VPD_leaf_). ɸ_E_ was determined as in Eq. 1. Data represent mean±se. VPD_leaf_ represents the mean at each level over all measurements. Different letters indicate significant differences between genotypes or groups at each VPD_leaf_ level (P<0.05; A>B>C>D). ^Ɨ^Groups as defined by k-means clustering of the gas exchange transpiration response (see Fig. 2). Note that genotypes were ordered by groups.

| **Genotype** | **Transpiration reduction ɸ_E_ (%)** | | | **Group^Ɨ^** | **Significance across groups**  **per VPD level** | | |
| --- | --- | --- | --- | --- | --- | --- | --- |
|  | ***2.69 kPa*** | ***3.28 kPa*** | ***3.87 kPa*** |  | ***2.69 kPa*** | ***3.28 kPa*** | ***3.87 kPa*** |
| Errans | 31.36±1.83 (A) | 42.79±1.49 (AB) | 50.90±1.62 (BC) | I | A | A | A |
| Balbisiana | 26.20±1.53 (AB) | 42.37±2.14 (AB) | 54.18±1.87 (ABC) | II | A | A | A |
| Zebrina | 30.97±3.18 (A) | 45.84±3.51 (A) | 59.43±2.80 (A) |  |  |  |  |
| Banksii_11 | 13.01±2.79 (C) | 25.89±3.04 (C) | 37.78±2.31 (D) | III | B | B | B |
| Banksii_17 | 19.10±2.72 (BC) | 33.28±2.89 (BC) | 44.05±2.57 (BCD) |  |  |  |  |
| Burmannica | 16.56±3.3 (BC) | 30.61±3.54 (BC) | 44.81±1.46 (CD) |  |  |  |  |
| Burmannicoides | 22.80±2.45 (ABC) | 35.19±2.37 (BC) | 44.03±5.10 (CD) |  |  |  |  |
| Malaccensis_33 | 14.55±3.59 (BC) | 24.57±3.96 (C) | 38.64±4.92 (D) |  |  |  |  |
| Microcarpa | 13.72±2.86 (C) | 24.73±3.51 (C) | 37.18±2.90 (D) |  |  |  |  |

**Table A.3:** Limitation of photosynthetic rate (A) at different leaf-to-air vapour pressure deficit (VPD_leaf_) levels. The limitation was determined as in Eq. 2. Data represent mean±se. VPD_leaf_ represents the mean at each level over all measurements. Different letters indicate significant differences between genotypes or groups at each VPD_leaf_ level (P<0.05; A>B>C). ^Ɨ^Groups as defined by k-means clustering of the gas exchange transpiration response (see Fig. 2). Note that genotypes were ordered by groups.

| **Genotype** | **Limitation of *A* (%)** | | | | | **Group** | **Significance across groups per VPD level** | | | | |
| --- | --- | --- | --- | --- | --- | --- | --- | --- | --- | --- | --- |
|  | ***1.50***  ***kPa*** | ***2.09***  ***kPa*** | ***2.69***  ***kPa*** | ***3.28***  ***kPa*** | ***3.87***  ***kPa*** |  | ***1.50***  ***kPa*** | ***2.09***  ***kPa*** | ***2.69***  ***kPa*** | ***3.28***  ***kPa*** | ***3.87***  ***kPa*** |
| Errans | 1.15  ±0.25 (A) | 2.27  ±0.49 (A) | 6.01  ±0.7 (A) | 10.38  ±0.73 (A) | 16.55  ±1.04 (A) | I | A | A | A | A | A |
| Balbisiana | 1.26  ±0.43 (A) | 1.42  ±0.44 (A) | 2.78  ±0.44 (B) | 5.77  ±0.33 (BC) | 10.9  ±0.8 (BC) | II | A | A | B | B | B |
| Zebrina | 1.45  ±0.3 (A) | 1.1  ±0.11 (A) | 2.64  ±0.42 (B) | 6.11  ±1.29 (B) | 12.38  ±2.27 (AB) |  |  |  |  |  |  |
| Banksii_11 | 1.15  ±0.12 (A) | 0.82  ±0.06 (A) | 1.64  ±0.28 (B) | 3.49  ±0.44 (BC) | 8.13  ±0.42 (BC) | II | A | A | B | C | C |
| Banksii_17 | 1.53  ±0.33 (A) | 1.2  ±0.28 (A) | 2.26  ±0.28 (B) | 4.81  ±0.56 (BC) | 9.74  ±0.87 (BC) |  |  |  |  |  |  |
| Burmannica | 1.05  ±0.3 (A) | 1  ±0.22 (A) | 2.31  ±0.13 (B) | 4.79  ±0.31 (BC) | 8.53  ±0.1 (BC) |  |  |  |  |  |  |
| Burmannicoides | 1.31  ±0.28 (A) | 2.06  ±0.08 (A) | 3.01  ±0.35 (B) | 4.94  ±0.4 (BC) | 8.95  ±0.4 (BC) |  |  |  |  |  |  |
| Malaccensis_33 | 1.39  ±0.69 (A) | 0.86  ±0.38 (A) | 2.04  ±0.89 (B) | 3.95  ±1.1 (BC) | 8.34  ±2.06 (BC) |  |  |  |  |  |  |
| Microcarpa | 2.53  ±0.92 (A) | 1.45  ±0.38 (A) | 1.42  ±0.47 (B) | 2.74  ±0.58 (C) | 6.65  ±0.84 (C) |  |  |  |  |  |  |

**Table A.4:** Stomatal reduction (ɸ_stom_) of leaf gas exchange measurements. ɸ_stom_ was determined as in Eq. 3. Data represent mean±se. Different letters indicate significant differences between genotypes or groups (P<0.05; A>B>C). ^Ɨ^Groups as defined by k-means clustering of the gas exchange transpiration response (see Fig. 2). Note that genotypes were ordered by groups.

| **Genotype** | **Stomatal reduction ɸ_stom_**  **(mol m^-2^ s^-1^ log_e_(kPa)^-1^)** | **Group^Ɨ^** | **Significance across groups**  **per VPD level** |
| --- | --- | --- | --- |
| Errans | 0.111±0.004 (C) | I | A |
| Balbisiana | 0.315±0.018 (A) | II | A |
| Zebrina | 0.160±0.034 (BC) |  |  |
| Banksii_11 | 0.113±0.021 (C) | III | A |
| Banksii_17 | 0.162±0.029 (BC) |  |  |
| Burmannica | 0.272±0.052 (AB) |  |  |
| Burmannicoides | 0.216±0.022 (ABC) |  |  |
| Malaccensis_33 | 0.174±0.037 (BC) |  |  |
| Microcarpa | 0.174±0.040 (BC) |  |  |

**Table A.5:** Whole-plant transpiration rate (E_rate_) response to step-increases in air vapour pressure deficit (VPD). Data represent mean±se values. VPD values represent the mean at each level over all measurements. Different letters indicate significant differences between genotypes (P<0.05; A>B>C>D).

| **genotype** | ***E_rate_ (g cm^-2^h^-1^)*** | | | | | | ***n*** | |
| --- | --- | --- | --- | --- | --- | --- | --- | --- |
|  | **VPD 0.77 kPa** | **VPD 1.36 kPa** | **VPD 1.93 kPa** | **VPD 2.34 kPa** | **VPD 2.64 kPa** |  | |  |
| Errans | 0.0082  ±0.0003 (C) | 0.0147  ±0.0007 (D) | 0.0163  ±0.0015 (C) | 0.0139  ±0.0018 (B) | 0.0132  ±0.0017 (B) | 8 | |  |
| Balbisiana | 0.013  ±0.0008 (AB) | 0.0218  ±0.0010 (AB) | 0.027  ±0.0013 (AB) | 0.0269  ±0.0014 (A) | 0.0269  ±0.0014 (A) | 6 | |  |
| Zebrina | 0.011  ±0.0004 (ABC) | 0.023  ±0.0005 (AB) | 0.0315  ±0.0011 (A) | 0.0328  ±0.0016 (A) | 0.028  ±0.0015 (A) | 5 | |  |
| Banksii_11 | 0.0118  ±0.0005 (AB) | 0.0198  ±0.0008 (BC) | 0.0267  ±0.0010 (AB) | 0.0301  ±0.0012 (A) | 0.0307  ±0.0014 (A) | 6 | |  |
| Banksii_17 | 0.0142  ±0.0008 (A) | 0.0241  ±0.0006 (A) | 0.0314  ±0.0020 (A) | 0.0327  ±0.0027 (A) | 0.032  ±0.0022 (A) | 4 | |  |
| Burmannica | 0.0124  ±0.0005 (AB) | 0.0205  ±0.0011 (ABC) | 0.0261  ±0.0018 (AB) | 0.0281  ±0.0026 (A) | 0.029  ±0.0032 (A) | 4 | |  |
| Burmannicoides | 0.01  ±0.0017 (BC) | 0.0173  ±0.0016 (CD) | 0.0248  ±0.0017 (B) | 0.0281  ±0.0017 (A) | 0.0284  ±0.0017 (A) | 6 | |  |
| Malaccensis_33 | 0.0129  ±0.0007 (AB) | 0.0227  ±0.0008 (AB) | 0.0303  ±0.0012 (A) | 0.0328  ±0.0015 (A) | 0.0323  ±0.0016 (A) | 6 | |  |
| Microcarpa | 0.0116  ±0.0015 (ABC) | 0.0213  ±0.0021 (AB) | 0.0269  ±0.0019 (AB) | 0.0304  ±0.0021 (A) | 0.0299  ±0.0020 (A) | 4 | |  |

**Table A.6:** Whole-plant transpiration reduction (ɸ_E_) at air vapour pressure deficits (VPD) of 2.64 kPa. ɸ_E_ was determined as in Eq. 1. Data represent mean±se. Different letters indicate significant differences between genotypes or groups at each VPD_leaf_ level (P<0.05; A>B>C>D>E>F). ^Ɨ^Groups as defined by k-means clustering of the gas exchange transpiration response (see Fig. 2). Note that genotypes were ordered by groups.

| **Genotype** | **Transpiration reduction ɸ_E_ (%)** | **Group^Ɨ^** | **Significance across groups per VPD level** |
| --- | --- | --- | --- |
| Errans | 54.43±5.38 (A) | I | A |
| Balbisiana | 35.12±1.19 (BC) | II | B |
| Zebrina | 40.49±1.59 (B) |  |  |
| Banksii_11 | 16.73±1.95 (EF) | III | C |
| Banksii_17 | 33.5±3.86 (BCD) |  |  |
| Burmannica | 21.31±5.1 (DEF) |  |  |
| Burmannicoides | 14.27±2.21 (F) |  |  |
| Malaccensis_33 | 27.02±1.58 (CDE) |  |  |
| Microcarpa | 28.52±4.54 (BCDE) |  |  |

**
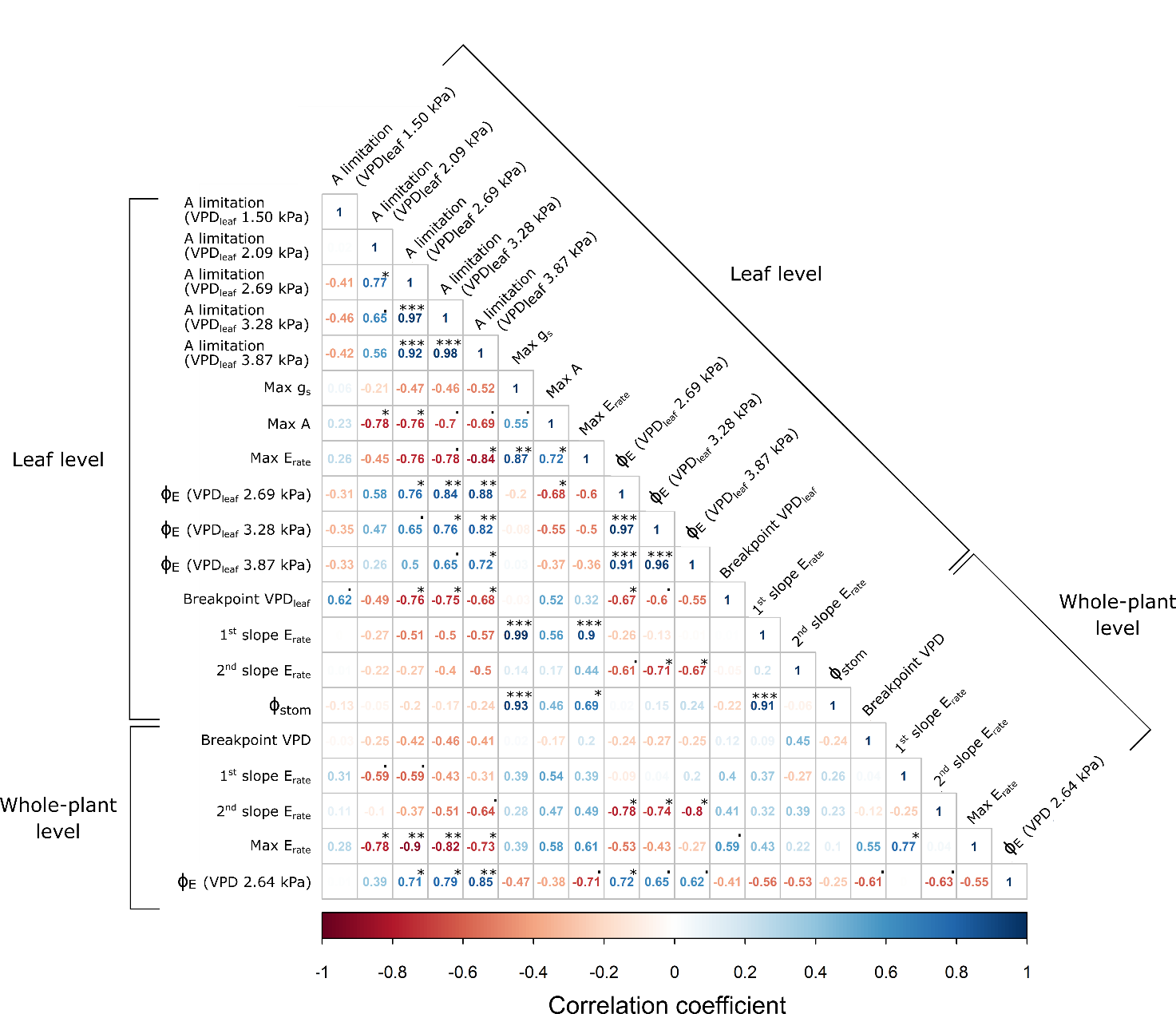
Fig. A.1:** Correlation matrix of the traits derived from the gas exchange response to step-increases in leaf-to-air vapour pressure deficit (VPD_leaf_) at leaf level and of the transpiration rate response to step-increases in air vapour pressure deficit (VPD) at whole-plant level. A limitation, percentage limitation of photosynthetic rate (*A*) at every VPD_leaf_ level; Max *g_s_*, maximum stomatal conductance (*g_s_*), max *A*; maximum *A*, max E_rate_, maximum transpiration rate; ɸ_E_, transpiration sensitivity at every VPD_leaf_ level; Breakpoint VPD_leaf_, breakpoint in transpiration rate with increasing VPD_leaf_; 1^st^ slope E_rate_, slope of transpiration rate before the breakpoint; 2^nd^ slope E_rate_, slope of transpiration rate after the breakpoint; ɸ_stom_, stomatal sensitivity. Red colours represent negative correlations, blue positive correlations. The distinction between traits measured at leaf level and whole-plant level is indicated by brackets (·P<0.10, *P<0.05, **P<0.01, ***P<0.001).
